# DIRAS2 as a key intermediate molecule joining with ferroptosis and neuronal excitation/inhibition (E/I) balance in epilepsy

**DOI:** 10.1016/j.gendis.2026.102374

**Published:** 2026-07-17

**Authors:** Chun Peng, Xiaoyuan Mao

**Affiliations:** a Department of Clinical Pharmacology, Hunan Key Laboratory of Pharmacogenetics and National Clinical Research Center for Geriatric Disease (Xiangya Hospital), Xiangya Hospital, Central South University, Changsha, Hunan 410008, China; b Institute of Clinical Pharmacology and Engineering Research Center of Applied Technology of Pharmacogenomics of Ministry of Education, Central South University, Changsha, Hunan 410078, China

Epilepsy remains one of the most prevalent severe neurological disorders, in which the chemical evidence lies in the imbalance of excitation/inhibition (E/I), ultimately resulting in neuronal hyperexcitability.^1^ Despite several investigations reporting the potential mechanisms triggering excessive neuronal excitation, which include abnormal neural circuits and glial structure dysfunction, the concrete molecular signaling mechanism is still unclear. In recent years, substantial evidence from diverse epileptic animal models and patients' tissue samples has proven the existence of ferroptosis, a novel regulatory cell death featured by iron-dependent accumulation of lipid peroxides and distinct from other types of cell death modalities like apoptosis, necroptosis, autophagy, and pyroptosis in the facets of genetics, morphology, and biochemistry.^2^ Furthermore, treatment with ferroptosis inhibitors alleviates seizure activity and neuronal injury,^3^ suggesting a critical role and a feasible therapeutic strategy for treating epilepsy via targeting ferroptosis. Indeed, the importance of ferroptosis for epilepsy also arises from three following aspects: first, the brain is a highly oxygen-consumed organ (approximately 20% of oxygen), which is susceptible to oxidative stress, a well-known contributing factor of ferroptosis; second, abundance of polyunsaturated fatty acids exist in neuronal membrane, a vital triggering factor of lipid peroxidation; third, iron and copper are rich within the brain, which help to catalyze the hydroxyl radical formation.^4^ Therefore, it is of vital importance to precisely identify the molecular mechanism to manipulate the ferroptotic process in epilepsy.

A recent publication in the *Proceedings of the National Academy of Sciences* by Tian's research group has illustrated that the brain-expressed GTP-binding RAS-like 2 gene (DIRAS2) acts as a ferroptosis-suppressing molecule and consequently ameliorates neuronal hyperexcitability via reducing the neuronal E/I ratio in a kainic acid-induced epilepsy mouse model and patients with temporal lobe epilepsy.^5^ Interestingly, in the kainic acid model, DIRAS2 displays a distinct dynamic expression pattern, with down-regulation during the acute/latent phase (1–7 days) but up-regulation during the chronic phase (28 days), consistent with its elevation in temporal lobe epilepsy patient tissues.^5^ Critically, this dynamic pattern—rather than the chronic phase elevation alone—highlights DIRAS2 as a pivotal therapeutic target tailored for early anti-epileptogenesis rather than merely a conventional anti-seizure treatment, offering a potential breakthrough for early intervention. Mechanistically, DIRAS2 suppressed neuronal ferroptosis via abrogating the extracellular signal-regulated kinase/p38 mitogen-activated protein kinase (MAPK) pathway in epilepsy (Fig. 1), and intervention with the specific ferroptosis inhibitor ferrostatin-1 promoted the restoration of neuronal E/I imbalance and epileptic phenotype caused by DIRAS2 knockdown.^5^ Traditionally, DIRAS2 is demonstrated to possess other versatile roles, such as protection of neurodevelopment and promotion of the cell cycle. Nowadays, Tian's research group uncovers a novel role of DIRAS2 linking ferroptosis, a critical molecular event under brain disorders like epilepsy. In this context, the DIRAS2–p38/MAPK–ferroptosis axis opens new avenues for therapeutic intervention. In the future, it is indispensable to explore DIRAS2-targeting small-molecule or gene therapies with a particular emphasis on early anti-epileptogenesis, which may also offer therapeutic potential for various other ferroptosis-sensitive or neuronal hyperexcitation-prone brain diseases, not limited to epilepsy.

**Figure 1:**
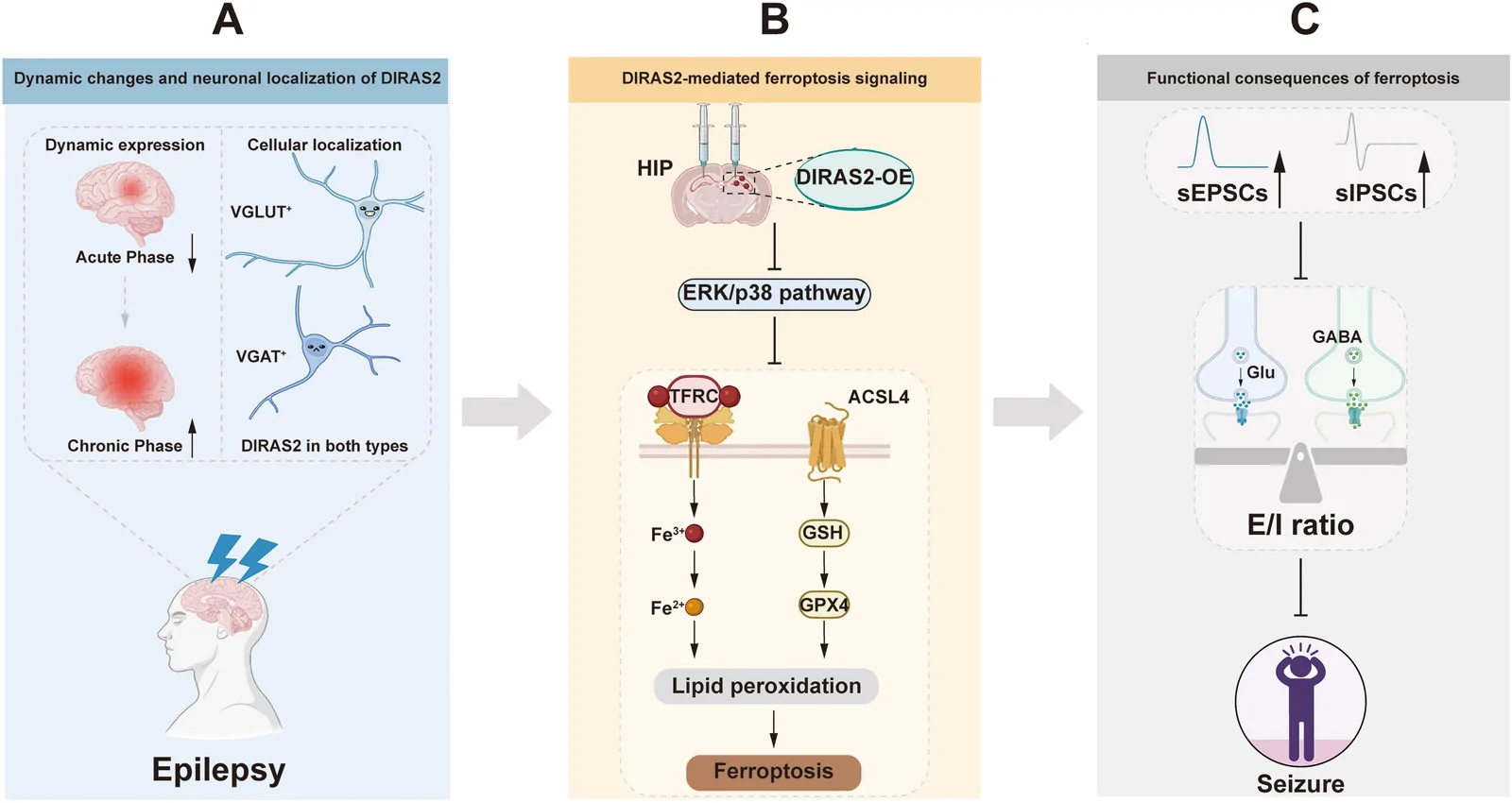
Proposed role of DIRAS2 in ferroptosis and excitation/inhibition (E/I) imbalance during epileptogenesis. In the kainic acid model, DIRAS2 exhibits dynamic expression changes during epileptogenesis and is present in both VGLUT^+^ and VGAT^+^ neurons. Mechanistically, DIRAS2 is proposed to suppress ERK/p38 MAPK signaling, leading to reduced TFRC and ACSL4-associated iron accumulation and lipid peroxidation. Through inhibition of ferroptosis, DIRAS2 may help restore E/I balance and thereby reduce seizure susceptibility.

## CRediT authorship contribution statement

**Chun Peng:** Writing – original draft. **Xiaoyuan Mao:** Writing – review & editing.

## Funding

This work was partially supported by the 10.13039/501100001809National Natural Science Foundation of China (No. 82474014, 82274027) and the Hunan Provincial Natural Science Foundation for Distinguished Young Scholars (China) (No. 2025JJ20087).

## Conflict of interests

The authors declare that they have no known competing financial interests or personal relationships that could have appeared to influence the work reported in this paper.
